# Supplementary material for: Are carbon emissions trading and green financial instruments synergistic? -Comprehensive quantitative research based on content analysis
Source: PLoS One. 2024 Mar 7;19(3):e0298601. doi: 10.1371/journal.pone.0298601 (PMC10919601; doi:10.1371/journal.pone.0298601)
Supplement: S1 Data — (ZIP) [file pone.0298601.s001.zip › Data/地级市Z_F工作报告.zip!/╔╜╢1⁄2/╢1⁄2╙¬╩╨/└┤╫╘╣1⁄2╓┌║┼í╛┬φ┐╦╩2╛▌═°í┐_2007.docx]

各位代表:

　　现在,我代表市人民政府向大会作政府工作报告,请予审议,并请市政协委员和其他列席会议的同志提出意见。

　　一、2006年工作回顾

　　过去的一年,是实施“十一五”规划的第一年。一年来,全市上下在省委、省政府和市委的正确领导下,以邓小平理论和“三个代表”重要思想为指导,全面贯彻党的十六大和十六届三中、四中、五中、六中全会精神,以科学发展观统领全局,认真执行国家宏观调控政策,同心同德,干事创业,经济社会发展取得了新的成绩。

　　国民经济平稳较快发展。初步统计,2006年全市实现生产总值1450.3亿元,比上年增长17%。工业发展速度和效益同步提高,全市规模以上工业实现增加值1107.6亿元,增长25.6%;利税769.8亿元,增长29.9%;利润541.7亿元,增长17%。高新技术产业产值占全市规模以上工业总产值的比重达到15.6%,提高2.1个百分点。服务业较快发展,实现增加值204.4亿元,增长20.8%。全社会固定资产投资完成609.9亿元,增长20.2%。实现社会消费品零售总额173.5亿元,增长16.5%;市场物价平稳,居民消费价格总水平上涨0.5%。民营经济规模扩大,个体工商户达到4.5万户、私营企业达到1.2万家,上缴税金增长42.7%。财政金融运行平稳,实现境内财政总收入361.68亿元,其中地方财政收入48.09亿元,分别增长30.85%和24.77%;各项存款余额761.6亿元,贷款余额516.4亿元,分别比年初增加109.7亿元和96.3亿元。

　　社会主义新农村建设扎实推进。积极培育水产、畜牧、蔬菜、冬枣、棉花等优势产业,培植农业龙头企业,发展农村合作经济组织,农业产业化经营水平进一步提高。突出抓好黄河口大闸蟹宣传推介,促进了农产品品牌战略的实施。实施千村万户农民科技培训工程,培训农民15.1万人次。加强农村基础设施建设,完成了麻湾三干等干支渠改造,建设改造农村公路712公里,农村集中供水覆盖到80万农民,沼气用户达到3万户,同时完成了300人以上村庄的规划编制,加强村庄改造和环境综合整治,改善了农民的生产生活条件和农村面貌。积极实施“万村千乡”市场工程和超市进乡镇、放心店进村工程,全市所有乡镇建立了超市,317个村建立了放心店,初步构建起农村现代商品流通服务体系。积极开展小康文明村创建活动,小康文明村达到328个。

　　改革开放迈出新步伐。招商引资继续推进,对外经贸进一步扩大,完成进出口总值18.8亿美元,其中出口11.2亿美元,分别增长26.8%和31.2%;吸收外商直接投资1.4亿美元,增长20.4%。各类园区发展较快,东营港经济开发区正式批准成立并启动建设。国有粮食购销企业改革全部完成,财税、国有资产管理、价格、事业单位等改革以及农村综合改革稳步推进。整顿和规范市场经济秩序,食品药品监管、打击商业欺诈、知识产权保护等工作取得新的成效。

　　城市建设得到加强。编制了新一轮城市总体规划以及一批中心城专业专项规划和重点区域详细规划。搞好中心城道路等市政设施建设和城区拆迁改造,加强环境综合整治,城市面貌得到改善。生态绿化建设项目获“中国人居环境范例奖”。推进县城和小城镇建设,全市城镇化率达到57.1%。

　　和谐社会建设富有成效。经济社会发展更加协调。农村义务教育经费保障机制基本建立,高中阶段教育加快发展,职业技术教育得到加强,教育质量有了新的提高。深化卫生监督和疾病防控体制改革,加强乡镇卫生院和社区卫生服务站建设,提高了疾病防控和医疗救治水平。推行医疗辅助检查“一单通”和单病种限价收费制度,减轻了群众就医负担。文化体育、新闻出版、广播电视事业健康发展,老龄、残疾人保障和妇女儿童工作进一步加强,双拥共建、国防教育、外事侨务、民族宗教、人民防空、无线电管理、气象、地震、史志、档案等各项事业都取得了新成绩。精神文明和民主法制建设得到加强,思想道德教育、创建文明城市、普法和依法治理等工作深入开展。积极推进“平安东营”建设,妥善化解各类社会矛盾,强化社会治安综合治理,毫不放松地抓好安全生产,保持了社会稳定。人口、资源、环境工作得到加强。全市人口自然增长率为4.15‰,人口和计划生育任务目标圆满完成。更加注重节能降耗,万元生产总值能耗降低4.5%,被命名为国家节水型城市。加强污染防治,加大环境保护和生态建设力度,二氧化硫排放量减少5.6%,化学需氧量排放量降低8%。强化促强扶弱措施,实施“突破利津”战略,深入开展油田帮扶乡镇工作,促进了区域经济社会协调发展。人民生活进一步改善。城市居民人均可支配收入达到16742元,增长12.1%;农民人均纯收入5157元,增长12%。着力解决事关群众利益的实际问题,加强就业再就业工作,城镇新增就业3.79万人,城镇登记失业率为1.93%。健全社会保障救助体系,“五保五救助”体系更加完善,城乡低保、农村“五保”供养标准进一步提高。首期市直经济适用房开工建设,新建农村特困户安居房1208套。认真解决征地、拆迁、改制中关系群众利益和农民工工资等问题,维护了群众的合法利益。为民办好十件实事,使广大群众得到更多的实惠。

政府自身建设取得新进展。实施依法行政“四五”规划取得新的成效。行政审批一站式服务和行政效能投诉监督机制逐步完善。实施公务员法工作扎实开展。电子政务建设进展加快,政府管理和服务的信息化水平明显提高。应急管理工作得到强化。不断完善廉政公开承诺制,加强教育监督管理,廉政建设和反腐败工作进一步加强。

　　各位代表,过去一年取得的成绩,是省委、省政府和市委正确领导的结果,是市人大及其常委会监督支持、人民政协和各民主党派参政议政的结果,是全市上下、油地军校、社会各界同心同德、艰苦奋斗的结果。在此,我代表市人民政府向全市人民和社会各界,向所有为东营的建设和发展做出贡献的同志们、朋友们,表示衷心的感谢并致以崇高的敬意!

　　回顾去年的工作,我们也清醒地看到,我市经济社会发展中还有不少问题,政府工作中也存在一些不足。主要是:产业结构单一,地方工业产业链条短、层次低,科技创新能力不强,服务业发展滞后;市属经济比较薄弱,缺乏产业支撑;城市建设缺少亮点,缺乏文化内涵,城市的影响力和吸引力不强;基础设施不够完善,特别是便捷的交通体系还未形成,人流物流集散能力差;生态比较脆弱,环境保护的压力大;影响社会和谐的因素较多,维护稳定的任务仍然很艰巨;政府职能转变不到位,政府工作人员在思想观念、驾驭市场经济能力、依法行政等方面还存在许多不适应的地方。对此,我们一定高度重视,采取切实措施,认真加以解决。

　　二、2007年工作的总体要求

　　2007年,是深入贯彻落实科学发展观、推进和谐社会建设的重要一年,也是实施“十一五”规划、站在新起点实现新发展的关键一年。综合分析国内外形势,我们既面临着良好的机遇,也面临着严峻的挑战。在新的一年里,我们要坚持以邓小平理论和“三个代表”重要思想为指导,深入学习贯彻党的十六大和十六届三中、四中、五中、六中全会精神,全面贯彻落实科学发展观,坚决贯彻国家宏观调控政策,全面实施黄河三角洲开发战略,着力调整经济结构和转变经济增长方式,着力加强资源节约和环境保护,着力推进改革开放和自主创新,着力促进社会发展和解决民生问题,促进经济又好又快发展,加快构建社会主义和谐社会,以新的作为、新的成绩迎接党的十七大胜利召开。

　　今年经济社会发展的主要预期目标是,全市生产总值增长12%,全社会固定资产投资增长18%,社会消费品零售总额增长15%,外贸出口总值增长17%,吸收外商直接投资增长13%,全市地方财政收入预算安排增长13%、工作目标增长15%,城市居民人均可支配收入增长10%,农民人均纯收入增长10%,城镇登记失业率控制在2%以内,居民消费价格总水平涨幅控制在3%以内。主要约束目标为,万元生产总值能耗降低4.5%,二氧化硫排放量减少8.3%,化学需氧量排放量降低5.6%,人口自然增长率控制在7.4‰以内。为了确保实现上述目标,工作中把握好“三个指导原则”和“一个总体战略”。

　　三个指导原则,一是,以科学发展观统领全局,促进经济又好又快发展。坚持科学发展,转变发展观念,创新发展模式,正确处理“好”与“快”的关系,着力在“好”上下功夫,切实做到发展速度、质量、效益相协调,消费、投资、出口相协调,人口、资源、环境相协调,努力提高经济增长的稳定性、协调性和可持续性。二是,认真落实国家宏观调控政策,大力推进经济结构调整。坚决维护国家宏观调控政策的统一性、权威性、有效性,正确处理“保”与“压”的关系,调整投资结构,加大向高新技术产业、先进制造业、服务业、城市公用基础设施、农村基础设施等方面的投资,以有效的增量带动经济结构调整和经济增长方式转变。三是,坚持以人为本,推进和谐社会建设。围绕解决人民群众最关心、最直接、最现实的利益问题,加快建立健全促进社会和谐的制度保障,完善与经济发展相适应的社会保障体系,加强社会公共服务体系建设,积极发展社会事业,加强社会管理和利益协调,努力化解社会矛盾,维护社会公平和正义,促进经济发展与社会进步、改善民生相统一,让人民群众共享改革和发展的成果。

　　一个总体战略,就是加快实施黄河三角洲开发战略,全面展开黄河三角洲高效生态经济区建设。确立这样一个总体战略,是经济社会发展客观规律的必然选择;是贯彻省委、省政府的重大决策部署,抢抓机遇的迫切需要;是东营历届领导班子的共同思索和广大人民群众的强烈意愿;是在具备了一定基础和必要条件下,站在新起点实现新发展,确立东营在山东省乃至环渤海地区战略地位的重大举措。我们要紧紧抓住这个总体战略,精心运筹,加快推进实施。在推进思路上,立足于干起来,尽快见到成效,以显著的成效赢得上级支持,大力造成快速崛起之势。在推进切入点上,着力于打造一流环境。兴衰系于发展,发展必重环境。环境决定着东营的未来。从环境切入,从文化旅游起步,建设黄河水城,开发黄河口旅游,丰富城市内涵,提升城市品位,塑造城市品牌。着力突破交通瓶颈,搞好港口、机场、铁路、高速公路建设,打开对外联系的大通道。以一流的环境聚集人气,聚合资源,发展高新技术产业,培育高效生态产业集群。在推进方式上,转变观念,开拓思路,搭建融资平台,推进招商引资,拓宽市场化运作的路子。在推进力量上,搞好大合唱,凝聚内力,激发全市人民的积极性和创造性,借助外力,充分发挥沿海城市开放的优势,以大开放促进大开发大发展。

各位代表、同志们!实施黄河三角洲开发战略,建设黄河三角洲高效生态经济区,是我们肩负的重大历史使命。我们务必增强责任感、紧迫感,抓住机遇而不可丧失机遇;我们务必集中精力,举全市之力,加快推进,一切方面都着眼于服从于服务于这个大局,一切工作都围绕实施这个总体战略展开;我们务必干好今年,高点起步,快速启动,展开布局,拉开科学发展、快速崛起的新格局。只要我们万众一心,奋力开拓,扎实工作,就一定能打开新局面,实现新跨越,开创东营美好的未来!

　　三、2007年工作的重点

　　(一)深入推进社会主义新农村建设。坚持把解决“三农”问题作为全部工作的重中之重,抓住发展生产、致富农民这个根本,促进农业不断增效、农村加快发展、农民不断增收。一是,大力发展现代农业。坚持用培育优势产业和实施品牌战略提升农业。在稳定粮食生产的同时,积极发展水产、畜牧、蔬菜、冬枣、棉花等优势产业,合理规划布局,引导优势产业聚集发展,集中建设一批特色鲜明的优势农产品基地。特别是抓好水产业,努力将其培育成在全省乃至全国有影响的产业。高度重视农产品质量安全,加快农产品质量标准和检验监测体系建设,扩大农业标准化生产规模,大力发展生态农业。加快实施品牌带动战略,抓好10大特优农产品的质量认定和商标注册、宣传推介,开展原产地保护,打响黄河口品牌。坚持用产业化形式经营农业。积极扶持壮大加工和销售龙头企业,发展行业协会和农村合作经济组织,今年重点扶持30家龙头企业发展壮大,围绕10大特优农产品组建行业协会,培育壮大农村合作经济组织。构建农产品营销网络,搞好产销对接,大力开拓市场。实施科技兴农工程,抓好10处优势农产品研发示范中心建设,加快新品种新技术的引进、试验、示范和推广。抓好10个试点乡镇农业服务体系建设和“三电合一”信息网络建设,整合资源,理顺体制,建立稳定的农村基层技术服务队伍。加大良种繁育、动植物疫病防治和农业机械化建设力度。二是,加强农村基础设施建设。实施农田水利基本建设工程,搞好麻湾四干等4条干渠节水改造,治理太平河等4条排河,开发和治理23万亩低产田。实施生态绿化工程,搞好干线公路、水系、村庄绿化和农田林网建设。实施农村沼气建设工程,新增沼气用户1万户。实施农村集中供水户户通工程,再解决10个乡镇和农场、424个村、27万群众饮水安全问题,实现农村集中供水的全面覆盖。实施公路建设改造工程,改造维修农村公路760公里。继续实施“万村千乡”市场工程和超市进乡镇、放心店进村“两进”工程,推动农村日用消费品、农业生产资料和农产品三大流通网络建设,为农民提供更好的流通和消费服务。实施人居环境建设工程,进一步改善农民生活条件和农村面貌。三是,努力提高农民素质。深入推进小康文明村建设,加大农民培训力度,努力培养有文化、有道德、懂技术、会经营的新型农民。依托农业龙头企业、农村合作经济组织、农资经营企业和农村党员干部现代远程教育网络,建立农民培训基地,培训农民10万人;选派农村技术骨干、农业龙头企业负责人到高等院校进行短期脱产培加 快建立农村投入稳定增长机制。今年财政支农资金的增量继续高于上年,固定资产投资用于农村的增量继续高于上年,土地出让收入用于农村建设的增量继续高于上年。认真落实粮食直补、良种补贴、农资综合补贴和农机购置补贴等支农惠农政策,进一步调动农民的生产积极性。加快培育农村金融市场,扩大农民小额贷款,鼓励更多的信贷资金投向农业和农村。　　(二)调整优化经济结构。推动工业上规模、上档次。一是,努力把现有支柱产业做大做强。加快用高新技术和先进适用技术改造化工、造纸、轮胎、纺织等产业,突出抓好华泰集团林纸浆一体化、万达集团全钢载重子午胎扩建等重点技改项目。推动产业整合,拉长产业链条,培育产业集群。二是,积极发展高新技术产业和先进制造业,培育形成新的支柱产业。围绕新材料、生物制药、电子信息三大高新技术产业和石油装备、汽车零部件等先进制造业,搞好招商引资,迅速引进一批大项目,同时强化工业和高新技术发展资金的引导作用,鼓励现有骨干企业和民间资本向这些领域发展。三是,大力培植骨干企业、名牌产品。支持华泰、科达、万达、利华益、方圆等30家重点骨干企业加快发展。鼓励大企业引进国内外战略投资者,支持石化、轮胎、纺织等企业加强联合,努力培育形成一批竞争力较强的企业集团。大力培育名牌产品和驰名商标,提高产品的附加值、竞争力和市场占有率。

加快发展服务业。发展服务业是调整产业结构、增加财政收入、扩大劳动就业的重要举措,是改善发展环境、聚集人气商气的有效途径。要把发展服务业摆上重要位置,加大推动力度,加快发展步伐。一是,突出发展文化旅游、交通物流、金融保险、休闲娱乐业。文化旅游业要突出黄河口、大油田、孙武故里、湿地生态特色,大力发展黄河口生态旅游、休闲观光旅游、油城旅游、文化旅游。重点搞好黄河口旅游开发,建设黄河口旅游度假区,与原始生态相辉映,打造旅游精品,拉动旅游开发,努力把东营建成旅游热点城市,争创中国优秀旅游城市。今年在搞好整体策划的基础上,着力推进实施游船码头和进入景区道路等建设项目,搞好生态观光、生态教育、湿地观鸟等旅游产品开发,启动创建国家4a级旅游景区工作。加快推进会展中心、文化艺术中心、体育中心等标志性文化设施建设,围绕现代传媒、演出娱乐、艺术培训、艺术品展销、创意设计等方面,培育一批文化龙头企业。交通物流业要依托港口、机场、铁路建设物流园区,培育物流市场,推进物流社会化,努力建设区域性物流中心。金融保险业要支持国有商业银行加快改革发展,培育壮大地方金融机构,积极吸引外地股份制银行和外资银行来我市设立分支机构,完善金融体系。创新金融服务产品,搞好银企合作,构建合作共赢的新型银企关系。继续完善保险业多元化竞争体制,大力拓宽保险业务渠道。休闲娱乐业要适应广大居民以及外来投资者的消费需求,开发建设不同层次的休闲娱乐项目,积极兴办集购物休闲、餐饮娱乐于一体的综合服务业项目。二是,改造提升商贸流通等传统产业。充分发挥百货大楼、商业大厦、东营银座、东营振华、蓝海等骨干企业的辐射带动作用,推动商贸流通企业设施改造、机制创新和经营方式转变,发展新型业态,提升现代化水平。三是,优化服务业发展环境。尽快编制出台服务业发展总体规划和行业发展专项规划,制定分行业扶持政策措施,综合运用财政、税收、金融、价格、土地等政策,引导服务业发展。制定实施服务业骨干企业培植计划,扩大服务业对外开放,打造一批骨干企业和服务业品牌。放宽和规范市场准入,吸引信贷资金、外资和民间资本进入服务业领域。努力扩大消费需求,积极培育消费热点,增加文化、旅游、健身、休闲消费。扩大农村消费。整顿和规范市场经济秩序,加强市场监管,严厉打击哄抬物价、制售假冒伪劣商品和商业欺诈等行为。

　　大力发展民营经济。进一步落实支持民营经济发展的各项政策措施,鼓励更多的人搞民营、办实业,壮大创业群体。引导民营企业搞好管理创新,加快发展壮大,提高竞争力。推进实施中小企业成长计划,对列入省、市成长计划的110家企业搞好扶持。加快建立健全融资担保、信用评价、技术信息、人才培训、法律服务、行业协会等六大服务体系。坚决治理“三乱”,切实保护民营企业的合法权益。

　　(三)推动区域经济协调发展。一是,促进县域经济又好又快发展。发展县域经济,是省委、省政府的重大战略部署,也是东营发展的关键所在。要进一步加大推动力度,支持县区发挥优势,培植特色产业,努力推进农业产业化、新型工业化和农村城镇化。强化促强扶弱措施,促进基础好的县区率先发展,基础差的尽快赶上,继续实施“突破利津”战略,营造县域经济竞相发展的良好局面。二是,加快市属经济发展。市属经济是城市竞争力的重要体现,是城市繁荣、文明、和谐的重要物质基础。市属经济薄弱,对县域经济起不到带动辐射作用,最终也将制约县域经济的发展。要加快壮大市属经济,实现市属与县域经济两个轮子一起转,互相促进、共同发展。突出抓好东营经济开发区和东营港经济开发区建设,运筹资金,加大投入,加快“两区”基础设施配套完善;坚持“项目立区”,真正引进一批“大高外”项目落户开发区,形成产业龙头,拉动市属经济发展,带动黄河三角洲大开发大开放。三是,大力支持胜利油田、石油大学、济军生产基地发展。胜利油田是我国第二大油田,是东营经济社会发展的重要支柱。全市上下要一如既往地大力支持油田的发展和改革,为其提供良好的社会环境和广阔的发展空间,使油田在区域经济社会发展中发挥更大作用。石油大学是国家“211工程”重点高校,为东营的经济社会发展培养了大批人才,提供了强有力的智力支撑。要为石油大学在东营进一步发展壮大、提高办学水平,提供有力的支持和良好的服务。济军生产基地与地方相互依存、共同发展,为东营经济社会发展做出了贡献。要继续支持济军生产基地在新的形势和条件下更好发展。要有效整合区域内资金、技术、人才、土地等资源,促进区域经济社会一体化发展。树立大市民观念,努力发展经济,建设城市,完善设施,加强管理,为包括油地军校干部群众在内的广大市民提供良好服务,创造良好的工作生活环境,共享东营的繁荣文明。

(四)塑造城市品牌和加强基础设施建设。围绕治理污染,拓宽挖深城市河道,形成“九横十纵”的主干水系网络,水路绿结合,塑造河湖相连、水绿共生、城水相依、舟楫相通的人与自然和谐的人居环境。今年抓好“四横一纵”主干水系和“两片”重点区域的规划建设,突出抓好广利河综合整治,形成一条城市景观走廊和文化长廊。同时,要完善体制,全面提高城市规划建设管理水平。以实施新一轮城市总体规划为契机,进一步加强各类规划编制工作,尤其是加大控制性详细规划和城市设计力度,加强规划监察,依法维护规划的严肃性。搞好城市道路等市政设施建设,推进旧城区和城中村改造。完善公交网络,合理布局公交线路和站点,更新车辆,延长运行时间,满足群众需求。进一步理顺城市管理体制,整合中心城区城管执法力量,明确市、区、街、居的城管职责,实现管理重心下移。推进数字化城市网络管理,强化社区和物业管理,全面提高城市管理水平。搞好县城和小城镇规划建设管理,加快城镇化进程。小城镇建设要因地制宜,突出特色,搞好规划,量力而行,逐步实施。深入开展环境综合整治,改善城乡面貌。

　　加强重大基础设施建设。加快东营港建设步伐,推进油码头和液体化工码头论证建设,搞好港口运营。在科学论证的基础上,搞好空港的改造升级,延长加厚跑道,扩建完善航站和航管设施,年内通过立项批复,开工建设。加快威乌高速公路东营段建设,争取黄大铁路尽早开工。规划建设火车站,今年开工建设道轨和站房楼。推进北部防潮堤建设,完善沿海防潮体系。

　(五)推进改革开放。围绕解决深层次矛盾和全局性关键问题,加快推进各项改革,为经济社会发展提供动力支持。一是,深化投资体制改革。制定完善投资体制改革配套措施,规范政府投资行为,加强政府投资项目管理,强化对项目招投标代理机构和招投标过程的监管,对非经营性政府投资项目推行代建制。组建城市建设投资发展有限责任公司,搭建融资平台,用市场机制推进经营性项目建设。进一步规范企业投资行为,严格按程序审批、核准和备案,严禁违规上项目。二是,深化企业改革。指导企业实施股权结构调整,完善法人治理结构,推进企业制度创新和管理创新。鼓励和支持企业通过规范改制实现境内外上市。支持油田存续企业改制。三是,深化农村改革。坚持农村基本经营制度,依法保护农民的土地承包权。加快推进乡镇机构、农村义务教育和县乡财政管理体制改革。继续推进农村林权和水利设施、渔港码头等产权制度改革。稳步推进化解乡村债务试点。规范农村“一事一议”,控制农资价格,防止农民负担反弹。四是,深化价格改革。稳步推进水、电、气等资源性产品价格改革,强化对垄断行业价格成本的监审,建立有利于资源节约和环境保护的价格形成机制,建立物价上涨与提高困难群众补贴标准联动机制。五是,推进行政管理体制改革。加快政企、政事、政府与中介机构分开。切实转变政府职能,推进政府管理创新,在搞好经济调节和市场监管的同时,完善社会管理制度,优化公共资源配置,加强公共设施建设,提高社会管理和公共服务水平。减少和规范行政审批事项,简化和规范审批程序。继续推进电子政务建设。六是,推进事业单位综合配套改革。加快经营性事业单位改企转制,改革社会公益类事业单位用人制度和分配制度,逐步理顺监督管理类事业单位管理体制。

　　扩大对外开放。着力提高“两个水平”。一是,提高招商引资水平。改进招商方式,着力抓好产业招商和专业招商,提高招商工作成效。坚持引进外资与促进产业优化升级相结合,引导外资更多地投向高新技术产业、先进制造业、现代服务业、基础设施和生态建设。完善调度协调机制,促进招商项目落地。二是,提高外经贸工作水平。调整优化出口产品结构,巩固扩大农产品、纺织品等传统产品的出口份额,大力开发高技术含量、高附加值的出口产品,努力扩大机电产品和高新技术产品出口,提高出口效益和竞争力。大力发展高水平的加工贸易型项目和企业,提高加工深度和效益。抓住有利时机,引导和鼓励企业扩大先进技术设备、重要资源和原材料进口。深入研究外经贸发展面临的新情况,引导企业提高应对市场变化和控制风险的能力。支持有条件的企业“走出去”发展,开展境外投资,开发紧缺资源,建立生产和销售基地,拓展发展空间。加强区域合作,主动对接青岛和烟台、融入半岛城市群,主动对接济南、加强与省会城市群经济圈的合作,主动对接天津滨海新区、融入环渤海经济圈,积极承接长三角、珠三角产业转移,充分发挥我市的比较优势,在区域合作中加快发展。

　　(六)突出抓好资源节约、环境保护和科技创新。建设资源节约型、环境友好型社会,是开发建设黄河三角洲高效生态经济区的题中应有之义和必然要求。生态环境关系东营的生存和发展。我们决不要高能耗的增长,决不要高污染的发展,我们不能吃子孙饭,而要为子孙后代留下碧水蓝天。要着力抓好化工、电力、建材、纺织、造纸等重点行业特别是50户重点企业的节能降耗。全面推进建筑节能。大力开发和推广应用先进节能技术,积极发展风能、太阳能等新型能源。积极发展循环经济,推行清洁生产,重点培育省级循环经济型示范园区和示范企业。加强土地节约集约利用。严格执行土地利用总体规划和年度计划,规范用地行为,严厉查处土地违法违规案件。清理闲置土地,盘活存量土地,加大土地开发整理复垦力度,搞好城镇建设用地增加与农村建设用地减少挂钩试点。高度重视水资源综合利用,抓好节水工作。加大环境整治力度。加强企业污染防治,重点抓好工业废水达标排放,搞好燃煤电厂和燃煤、燃油锅炉脱硫设施改造。加强重点区域环境治理,抓好中心城生活污水处理和垃圾无害化处理设施改造建设,加快各县区、重点镇污水和垃圾处理设施建设。切实加大矿区环境治理力度。抓好小清河等流域综合治理,搞好海洋环境保护。加强自然保护区、饮用水源地等重要生态功能保护区的保护。实施农村小康环保行动计划,搞好农村的污染治理。积极开展创建生态县、环境优美乡镇、环境友好企业和文明生态村活动,大力推进生态市建设。加强节能环保政策促进体系建设,制定实施严格的能耗标准和污染物排放标准,制定完善有利于节能环保的财政、税收、价格政策。严格执行环境影响评价和“三同时”制度,认真落实排污总量控制和排污许可证制度。完善能耗和环境信息公报制度。建立节约集约用地考核评价体系和管理办法,把节约集约用地工作纳入政府目标责任制。

　　大力推进科技创新。完善创新扶持政策,搞好科技资源整合,健全科技创新体系,提高企业技术创新能力,建设创新型城市。加快推进石油大学科技园建设,将其建成辐射带动力强的科技创业创新平台。充分发挥企业主体作用,鼓励支持企业加大研发投入,加强技术研发中心建设,同时依托骨干企业组建一批行业技术研发中心。引导企业加强应用技术和高新技术研究,深化产学研结合,抓好先进技术的引进、消化、吸收和再创新,加快科技成果的转化应用。完善人才政策,优化人才环境,努力培养引进一批高层次人才,依托职业技术院校培养一批高技能人才。

　　(七)做好财税工作。加强财源建设,强化税收和各种非税收入征管,确保财政收入稳定增长。合理配置财政资源,调整优化支出结构,重点保工资、保运转、保法定支出、保公益性事业和社会保障支出,建设资金主要靠市场化运作。深化财政体制改革,创新和完善预算编制、政府采购、国库集中收付和收支两条线等管理制度,规范财政转移支付制度,继续搞好“金财工程”建设,推进财政精细化管理。搞好国有资产管理和运营,盘活有效资产,实现保值增值。坚持理财与调整结构、发展经济相结合,与生财聚财相结合,与解决民生问题相结合;坚持财政资金统一管理,集中财力办大事;坚持依法办事,规范财政资金管理使用,不断提高科学理财水平。大力提倡勤俭节约,改进公务接待。进一步强化审计监督,坚决查处违规违纪问题,维护良好的财经秩序。

　　(八)促进社会事业发展和社会文明进步。巩固提高基础教育,大力发展职业技术教育,积极发展高等教育,继续发展学前教育,促进各级各类教育协调发展。加大教育投入,进一步完善农村义务教育经费保障机制,继续改善中小学办学条件。搞好区域教育规划,推动城区教育资源整合,调整学校布局。加快东营职业学院和东营市技术学院等职业技术院校发展。大力推进素质教育,加强教师队伍建设,提高教育质量。强化政府责任,加大政府投入,逐步建立覆盖城乡居民的基本卫生保健制度。在城区继续推进以社区卫生服务中心、服务站为主体的服务网络建设,完善服务功能,扩大覆盖面。在农村实行医防分类管理,加强乡镇卫生院的急救能力和服务网点建设。深入开展山东省公共卫生安全示范区创建活动,加强疫病防控体系建设,加大防控力度,提高公共卫生服务水平和突发公共卫生事件应急处置能力,确保公共卫生安全。深化医疗卫生体制改革,规范医疗服务、药品生产流通秩序,大力实施惠民医疗工程,逐步解决群众看病难、看病贵问题。坚持文化事业、文化产业一起抓,加快公益性和基层文化设施建设,完善公共文化服务体系,不断创新文化事业管理机制和文化产业经营机制,增强文化发展活力,活跃城乡文化生活。努力提高竞技体育水平,广泛开展群众体育活动。发展广播电视、新闻出版事业。关心支持妇女儿童、残疾人、老年人工作。坚持不懈地抓好人口和计划生育工作,稳定低生育水平,提高出生人口素质。加强全民国防教育,抓好民兵预备役建设,巩固扩大双拥共建成果。进一步做好外事侨务、民族宗教、无线电管理、档案、气象、对台、地名、史志、人防、防震和减灾救灾等各项工作。加强精神文明建设,深入开展思想道德教育和社会主义荣辱观教育,大力建设和谐文化,广泛开展群众性精神文明创建活动,积极推进创建文明城市、“信用东营”建设等工作。加强民主法制建设,搞好“五五”普法教育,推进依法治理。各级政府要自觉接受人大及其常委会的监督,认真执行人大及其常委会的决议决定,支持人民政协履行政治协商、民主监督、参政议政职能,认真办理人大代表建议和政协委员提案,加强与民主党派、工商联、人民团体和社会各界的联系。扩大基层民主,保障群众依法行使民主权利。

　　(九)认真解决群众切身利益问题。把扩大就业作为经济社会发展的重要目标,推动经济发展和就业良性互动。认真落实促进就业再就业的优惠扶持政策,鼓励发展就业容量大的产业,支持自谋职业、自主创业,有效增加就业岗位。健全再就业援助机制,积极开发公益性就业岗位,着力帮助零就业家庭和困难群体就业。统筹做好城镇新成长劳动力、被征地农民、高校毕业生和复员转业军人等群体的就业工作。今年确保城镇新增就业3万人以上,农村转移劳动力3万人。大力推进和谐劳动关系建设,规范企业用工行为,全面实行劳动合同制度和集体协商制度,健全劳动保障监察体制和劳动争议调处仲裁机制,依法及时处理劳动争议。建立健全与经济发展相适应的收入增长机制,不断提高居民收入水平。严格执行最低工资制度,建立农民工工资支付保障体系。围绕“十一五”规划目标,进一步健全完善社会保障救助体系,巩固城乡“五保五救助”成果,逐步提高保障标准,增强保障能力。加快建立面向进城务工农民和失地农民的社会保障制度,推行新型农村养老保险,做好非公有制企业和城镇灵活就业人员参保工作,扩大社会保障覆盖面。加大社会保险费征缴力度,多渠道筹集社保基金,完善监管制度,确保基金安全和保值增值。搞好经济适用房建设,建立和完善廉租房制度。继续实施农村特困群众安居工程。积极发展社会福利和慈善事业。认真解决土地承包、土地征用、房屋拆迁、企业改制等涉及群众利益的问题。加强食品药品监管,确保群众饮食用药安全。从群众的愿望出发,扎扎实实为群众办实事、解难题。

　　(十)切实维护社会稳定。深入推进“平安东营”建设,健全社会利益表达、协调、引导和基本利益保障机制,满足不同群体的合理利益诉求。健全信访工作责任制和矛盾纠纷排查调处机制,认真解决群众反映的突出问题,及时化解各种社会矛盾。强化社会和油区治安综合治理,加强基层基础工作,加强社会治安防控体系建设,搞好社区和农村警务室建设,加强出租房屋和流动人口管理。深入开展严打整治特别是打黑除恶专项斗争,坚决打击各种违法犯罪行为。高度重视安全生产,严格落实安全生产责任制,强化企业主体责任,深入开展专项整治,加强安全生产监管,坚决遏制重特大事故的发生。完善应急管理体制,形成统一指挥、反应灵敏、协调有序、运转高效的应急管理机制,有效应对自然灾害、事故灾难、社会安全事件,提高危机管理和抗风险能力。

　　四、加强政府自身建设

　　全面落实科学发展观、构建和谐社会,加快实施黄河三角洲开发战略,对政府自身建设提出了新的更高的要求。面对新形势、新任务、新机遇、新挑战,必须认真开展“解放思想,改进作风,全面落实科学发展观”教育活动,着力“破旧、克满、戒躁、纠散、反奢、防片”,以此推动政府自身的改革和建设,不断提高工作水平。

　　强化科学发展和市场经济观念,提高推动发展的能力。深刻理解和把握科学发展观的精神实质,增强贯彻落实的自觉性,以科学发展的理念指导发展,以科学的思维方式谋划发展。坚持与时俱进,克服因循守旧、自满自足;坚持按经济规律办事,克服违背规律、盲目蛮干;坚持统筹兼顾,克服只看眼前、不顾长远;坚持少说多做,克服做表面文章、搞花架子;坚持低调务实,克服好大喜功、脱离实际,打开促进科学发展的思想通道。把发展的热情、科学的态度和务实的作风统一起来,切实使各项工作更加经得起历史、实践和群众的检验。改进工作方式和管理方式,打破传统的思维定式和工作模式,学会并善于市场化运作。全体政府工作人员都要加强学习,善于学习,不断丰富头脑,开阔视野,积累经验,增长本领,以新的境界、新的作为推动发展。

　　强化依法行政观念,推进法治政府建设。深入贯彻《全面推进依法行政实施纲要》,进一步实施我市依法行政“四五”规划。各级政府及部门必须依法行使权力,履行职责,接受监督,克服工作中的随意性和无序性。凡是政府应该履行的职责,特别是社会管理、公共服务,一定要做到位,不能不作为;应该由市场来调节和配置资源的,一定要由市场来运作,不能乱作为。深化行政执法体制改革,进一步明确执法权限,完善执法程序,提高执法水平。全面推行行政执法责任制,加强行政复议和执法监督,完善执法评议考核、行政赔偿和过错追究制度,认真处理和纠正行政不作为、滥用职权、执法不公、以权谋私等行为。大力推行政务公开,畅通政府公报、政府网站、新闻发言人和政府公开电话等渠道,保障人民群众对政府工作的知情权、参与权和监督权。进一步健全重大事项集体决策、专家咨询、社会公示与听证、决策评估等制度,实行依法决策、科学决策、民主决策,加强政府诚信建设,增强政府的执行力和公信力。

　　强化高效务实和整体配合观念,狠抓工作落实。树立正确的政绩观,求真务实,说实话、办实事、求实效。树立强烈的事业心、责任感,艰苦奋斗,戒骄戒躁,扎实工作,埋头苦干。树立良好的精神状态,振奋精神,锐意进取,争创一流业绩。改进会风文风,少开会、开短会,讲短话、发短文,最大限度地减少礼仪性、应酬性活动,深入实际、深入基层、深入群众,调查研究,解决问题。完善目标责任制,强化政务督查考核,严格奖惩,确保各项工作落到实处。对今年确定的便民实事、重要工作、重点项目,都要明确责任,落实分管领导和责任部门,一抓到底,确保完成。政府部门一定要把握大局、维护大局,恪尽职守,团结协作,配合联动,切实克服各自为政、分散主义、低效扯皮等问题。要严明纪律,认真贯彻执行上级的决策部署,令行禁止,确保政令畅通。

　　强化执政为民观念,推动廉政建设。我们的政府是人民的政府,政府的权力是人民赋予的。要自觉践行“三个代表”重要思想,坚持人民的利益高于一切,真正做到立党为公、执政为民。坚持从严治政,加强领导干部作风建设,大力倡导“八个方面的良好风气”。建立健全教育、制度、监督并重的惩治和预防腐败体系,不断拓展从源头上防治腐败工作领域,把反腐倡廉工作推向深入。注重加强制度建设,建立健全项目招投标、土地招拍挂、政府采购、政府投资项目管理、财政资金管理、国有资产管理、城市规划审批、人事编制管理等各项制度,切实做到照章办事。进一步抓好治理商业贿赂工作,建立健全长效机制。全体政府工作人员一定要加强思想道德修养,廉洁从政,遵纪守法,特别是各级领导干部要牢记“两个务必”,率先垂范,廉洁自律,一身正气,两袖清风。继续加大案件查处力度,对违法违纪行为,不论涉及到谁,都要一查到底,决不手软。

　　各位代表、同志们!奋斗孕育发展,开拓成就伟业。让我们紧密团结在以胡锦涛同志为总书记的党中央周围,在党中央国务院、省委省政府和市委的坚强领导下,高举邓小平理论和“三个代表”重要思想伟大旗帜,全面贯彻落实科学发展观,加快实施黄河三角洲开发战略,奋发有为,开拓进取,为建设繁荣、文明、和谐的新东营,为东营人民更加富裕、幸福、安康而努力奋斗!
